# The Function of CD3^**+**^CD56^**+**^ NKT-Like Cells in HIV-Infected Individuals

**DOI:** 10.1155/2014/863625

**Published:** 2014-03-20

**Authors:** Yongjun Jiang, Xiaojian Cui, Chen Cui, Jian Zhang, Fangyuan Zhou, Zining Zhang, Yajing Fu, Junjie Xu, Zhenxing Chu, Jing Liu, Xiaoxu Han, Christina Liao, Yanan Wang, Yaming Cao, Hong Shang

**Affiliations:** ^1^Key Laboratory of AIDS Immunology of National Health and Family Planning Commission, Department of Laboratory Medicine, The First Affiliated Hospital, China Medical University, No. 155 Nanjing North Street, Heping District, Shenyang 110001, China; ^2^Collaborative Innovation Center for Diagnosis and Treatment of Infectious Diseases, Hangzhou 310003, China; ^3^Tianjin Children's Hospital, Tianjin 300074, China; ^4^Department of Immunology, Basic Medical College, China Medical University, Shenyang 110001, China

## Abstract

CD3^+^CD56^+^ NKT-like cells are one of the critical effectors in the immune response to viral infection and tumors, but the functional features of NKT-like cells in HIV infection have been rarely reported. In this study, we observed and described the state of NKT-like cell functions in primary HIV-infected individuals (PHIs), chronic HIV-infected individuals (CHIs), long-term nonprogressors (LTNPs), and HIV-negative controls (NCs). The results showed that the percentage of IFN-**γ** ^+^CD3^+^CD56^+^ NKT-like cells was notably higher in LTNPs compared with CHIs, and the proportion of CD3^+^CD56^+^ NKT-like cells with dual function (IFN-**γ** ^+^CD107a^+^ NKT-like cells) in LTNPs was also much higher than in CHIs. Additionally, the percentages of IFN-**γ** ^+^CD107a^+^ NKT-like cells negatively correlated with viral load. Taken together, our data demonstrated that good functions of CD3^+^CD56^+^ NKT-like cells in LTNPs likely occurred as a protective mechanism that slows down HIV disease progression.

## 1. Introduction 

CD3^+^CD56^+^ NKT-like cells are a broader group of T cells that conform to the original definition of NKT cells, which is “T cells coexpressing NK cell markers” [1–3]. Innate CD3^+^CD56^+^ NKT-like cells comprise approximately 5 to 15% of the peripheral T-cell pool and up to 50% of T cells within the liver environment [4]. Due to repeated antigen exposure, CD3^+^CD56^+^ NKT-like cells gradually increase in absolute number in older individuals but are absent in cord blood [5].

Unlike classical T cells, CD3^+^CD56^+^ NKT-like cells possess both innate and adaptive immune functions, displaying capabilities of both T and NK cells. That is, they can carry out both major histocompatibility complex- (MHC-) restricted and MHC-unrestricted cytotoxicity and secrete many kinds of cytokines [2, 6–8]. Activation of CD3^+^CD56^+^ NKT-like cells may be crucial because of their ability to serve as an early source of regulatory cytokines and their degranulation-related killing function. Regulatory cytokines, such as interferon-*γ* (IFN-*γ*), are known to induce antiviral responses and shape the Th1 immune response [9]. Furthermore, CD3^+^CD56^+^ NKT-like cells share several characteristics with NK cells, such as the large granular lymphocyte morphology, the capacity to lyse NK-sensitive target cell line, and the activities regulated by inhibitory KIR and CD94 molecules [10, 11]. As CD3^+^CD56^+^ NKT-like cell degranulation occurs, secretory lysosomes are released, and lysosome-associated membrane protein-1 (LAMP-1, CD107a) is transported to the surface of cells to act as a functional marker.

Their dual functions place CD3^+^CD56^+^ NKT-like cells alongside natural killer T cells as frontline innate immune effectors and potential regulators of adaptive immune responses against microorganisms. Numerical and functional deficiencies as well as phenotypic and functional alterations of CD3^+^CD56^+^ NKT-like cells have been reported in patients with various infections, autoimmune diseases, and cancers. For example, in the livers of chronically hepatitis C virus- (HCV-) infected individuals, reported numbers of CD3^+^CD56^+^ NKT-like cells are remarkably low, which may be related to susceptibility for hepatocellular carcinoma [12]. In individuals with hematopoietic malignancies who receive hematopoietic stem cell transplants, the functional mature CD3^+^CD56^+^ NKT-like cells may produce cytotoxicity against cancer cells and play a therapeutic role against hematopoietic malignancy [13]. Studies suggest that the cytotoxic function of CD3^+^CD56^+^ NKT-like cells in patients with human ovarian cancer or prostate cancer was obviously impaired [8]. Additionally, the low percentage of CD3^+^CD56^+^ NKT-like cells was associated with a higher death risk in chronic lymphocytic leukemia (CLL) patients [14].

CD3^+^CD56^+^ NKT-like cell activity has been reported in the activation and regulation of multiple arms of the immune response while reports on its function in HIV infection are rare. Montoya et al. found that expression of IFN-*γ* by PMA/ionomycin-activation in CD3^+^CD56^+^ NKT-like cells was significantly higher in exposed HIV-seronegative individuals (ESN) than in controls. This finding suggested that IFN-*γ* production by CD3^+^CD56^+^ NKT-like cells might be a factor involved in preventing sexually transmitted HIV-1 infection [15]. But whether the function of CD3^+^CD56^+^ NKT-like cells changes during primary HIV infection or significantly impacts the rate of disease progression has not been determined. Therefore, studying CD3^+^CD56^+^ NKT-like cell function may have important implications for understanding the mechanisms that counter HIV infection.

In this study, we investigated the characterization of CD3^+^CD56^+^ NKT-like cell function in primary HIV-infected individuals (PHIs), chronic HIV-infected individuals (CHIs), and long-term nonprogressors (LTNPs) and also analyzed the relationship of CD3^+^CD56^+^ NKT-like cell function with HIV disease progression.

## 2. Materials and Methods

### 2.1. Study Population

The 30 HIV-infected individuals who were chosen to participate in this study were males, aged 22 to 53 (33 ± 10) and included PHIs, CHIs, and LTNPs. PHIs were defined as individuals who had been HIV-positive for less than 3 months. LTNPs were defined as HIV-infected individuals who had been asymptomatic for 10 years or more, whose CD4^+^ T cell counts were greater than 500/*μ*L in the absence of antiretroviral therapy. CHIs were defined as HIV-infected individuals who had been infected with HIV for more than 3 months and less than 8 years without antiretroviral therapy. According to these criteria, 10 PHIs were enrolled. They had a median CD4^+^ T cell count of 484 cells/*μ*L (range: 166 to 774 cells/*μ*L) and a median viral load of 52,000 copies/mL (range: 728 to 10^7^ copies/mL). Additionally, 13 CHIs were enrolled who had a median CD4^+^ T cell count of 328 cells/*μ*L (range: 240 to 986 cells/*μ*L) and a median viral load of 30,903 copies/mL (range: 40 to 489,779 copies/mL). Finally, 7 LTNPs were recruited for the study. They had a median CD4^+^ T cell count of 704 cells/*μ*L (range: 547 to 1000 cells/*μ*L) and a median viral load of 562 copies/mL (range: 40 to 5623 copies/mL). For the normal controls (NCs), 14 HIV-negative males between ages 22 and 60 (31 ± 8) were recruited. These NCs also met the following criteria: HIV-antibody-negative; normal blood cell counts, hemoglobin level, and liver function; and no history of immunologic disease. The Research and Ethics Committee of the First Affiliated Hospital of China Medical University approved the study, and each individual gave written informed consent for participation in the study (Table 1).

### 2.2. Assessment of NKT-Like Cell Functions

**Figure 1:**
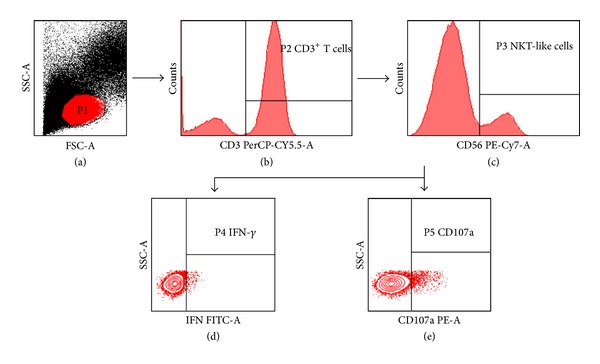
Gating strategy. P1 gate is lymphocytes (a), P2 gate is CD3^+^ lymphocytes (b), P3 gate is CD3^+^CD56^+^ NKT-like cells (c), and the expressions of IFN-*γ* and CD107a, in NKT-like cells are further analyzed ((d) and (e)).

The functions of NKT-like cells in PBMCs were detected after coincubation with the K562 cell line or PMA/ionomycin. Because NKT-like cells share some features with NK cells, they can recognize HLA class I-negative cell line K562 and their functions can be detected through coculture with K562 [10, 11]. PBMCs were coincubated for six hours with K562 cell line at an E : T ratio of 5 : 1. Meanwhile, PBMCs were stimulated with PMA (Sigma, Cat. No. P-8139, USA) and ionomycin (Sigma, Cat. No. I-0634, USA) in final concentrations of 50 ng/mL and 1 *μ*M, respectively. PE-conjugated anti-CD107a (BD Biosciences, USA) and monensin (Becton-Dickinson, USA) were added to all incubated samples. Then cells were stained with both Percp-conjugated anti-CD3 and PE-cy7-conjugated anti-CD56 (BD Biosciences, USA). The cells were made permeable using Perm/Wash (Becton-Dickinson, USA) for 10 minutes, stained with FITC-conjugated anti-IFN-*γ* (BD Biosciences, USA) for 30 minutes at 4°C, washed, and then fixed in 1% formaldehyde. NKT-like cell populations were defined by dual-positive expressions of CD3 and CD56 molecules. The frequency of IFN-*γ* and CD107a expression in NKT-like cells were quantified by multicolor flow cytometry (Figure 1).

### 2.3. Determination of CD4^+^ T Cell Counts

CD4^+^ T cell counts were measured by flow cytometry (FACS Calibur, Becton-Dickinson, USA). A single-platform lyse-no-wash procedure was performed using Trucount tubes and TriTEST anti-CD4-FITC/CD8-PE/CD3-PerCP reagents (Becton Dickinson, USA). Trucount Control Beads (low, medium, and high beads; Becton Dickinson, USA) were used to control the quality and accuracy of the CD4^+^ T cell true count test.

### 2.4. Measurement of HIV Viral Loads

Plasma HIV RNA was measured via RT-PCR using the COBAS AmpliPrep/COBAS Taqman (Roche Diagnostic Systems). The detection range of the assay was between 40 copies/mL and 10,000,000 copies/mL. HIV RNA copy numbers were calculated according to the manufacturer's reference standards.

### 2.5. Statistical Analysis

The nonparametric Mann-Whitney *U* tests were used for comparisons between two groups. Correlations between variables were evaluated using the Spearman's rank correlation test. All analyses were carried out using SPSS 17.0 software and *P* values <0.05 were considered significant.

## 3. Results 

### 3.1. Changes in the Functional Activity of NKT-Like Cells during HIV-1 Infection

**Figure 2:**
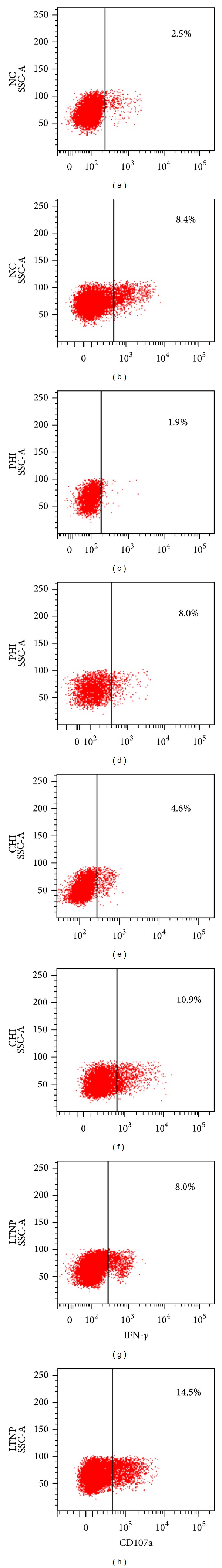
Representative flow cytometric graphs of NKT-like cells production of IFN-*γ* and expression of CD107a in NC group ((a) and (b)), PHI group ((c) and (d)), CHI group ((e) and (f)), and the LTNP group ((g) and (h)).

NKT-like cells in peripheral blood mononuclear cells (PBMCs) were cultured with the MHC null K562 cell line, and the functional activities of NKT-like cells in NCs were compared with the HIV-infected groups, including PHIs, CHIs, and LTNPs. Functional activity was assessed by measuring production of IFN-*γ* cytokine and expression of CD107a protein. Representative flow cytometry figures are shown in Figure 2.

**Figure 3:**
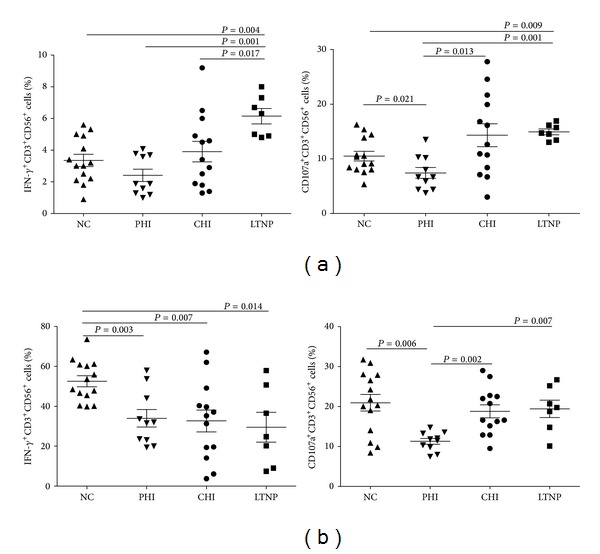
Functional activity of NKT-like cells was compared in NCs, PHIs, CHIs, and LTNPs. Functional activity was assessed by measuring IFN-*γ* ^+^CD3^+^CD56^+^ cells% and CD107a^+^CD3^+^CD56^+^ cells%. (a) NKT-like cells in PBMC were cultured with the MHC null K562 cell line; (b) NKT-like cells in PBMCs were coincubated with the strong stimulation of PMA/ionomycin. Data were analyzed by the Mann-Whitney *U* tests and *P* values <0.05 were considered significant.

We found that percentage of IFN-*γ* ^+^CD3^+^CD56^+^ was higher in LTNPs than that in NCs and PHIs (*P* = 0.004 and *P* = 0.001, resp.). It was also higher in LTNPs than that in CHIs (*P* = 0.017). The percentage of CD107a^+^CD3^+^CD56^+^ was lower in PHIs than that in NCs, CHIs, or LTNPs (*P* = 0.021, *P* = 0.013, and *P* = 0.001, resp.). NKT-like cells in LTNPs expressed more CD107a compared with NCs (*P* = 0.009) but had the similar CD107a expression as CHIs (Figure 3(a)).

After using K562 cell line to test NKT-like cell functions, we also used the strong stimulation of PMA/ionomycin to determine the functions of NKT-like cells. It was found that the percentage of IFN-*γ* ^+^CD3^+^CD56^+^ was significantly higher in NCs than that in any of the HIV-infected individuals (*P* = 0.003, *P* = 0.007, and *P* = 0.014, resp.). Compared with NCs, CHIs, and LTNPs, NKT-like cells in PHIs expressed less CD107a (*P* = 0.006, *P* = 0.002, and *P* = 0.007, resp.) (Figure 3(b)).

### 3.2. Combined Analysis of the Functional Activity of NKT-Like Cells during HIV Infection

**Figure 4:**
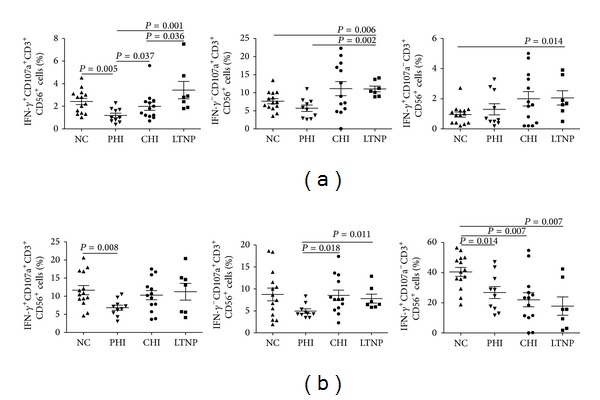
Combined analysis of the functional activity of NKT-like cells in NCs, PHIs, CHIs, and LTNPs. Combined analysis of the functional activity of NKT-like cells was assessed by measuring IFN-*γ* ^+^CD107a^+^ NKT-like cells%, IFN-*γ* ^−^CD107a^+^ NKT-like cells%, and IFN-*γ* ^+^CD107a^−^ NKT-like cells%. (a) NKT-like cells in PBMC were cultured with the MHC null K562 cell line; (b) NKT-like cells in PBMCs were coincubated with the strong stimulation of PMA/ionomycin. Data were analyzed by the Mann-Whitney *U* tests and *P* values <0.05 were considered significant.

We performed a combined analysis of the IFN-*γ* production and CD107a expression when PBMCs were cultured together with the MHC null K562 cell line. We found that the percentage of IFN-*γ* ^+^CD107a^+^ NKT-like cells in the LTNP group was higher than in the CHI group (*P* = 0.036) and in the PHI group (*P* = 0.001). Compared with those of other groups, NKT-like cells produced less IFN-*γ* and displayed lower CD107a expression (*P* = 0.005, *P* = 0.037, and *P* = 0.001, resp.) in PHIs. The proportion of IFN-*γ* ^−^CD107a^+^ NKT-like cells in LTNPs was also higher than that in NCs and PHIs (*P* = 0.006 and *P* = 0.002). The percentage of IFN-*γ* ^+^CD107a^−^ NKT-like cells in LTNPs was higher than that in NCs (*P* = 0.014) (Figure 4(a)). The results inferred that LTNP had good activities in NKT-like cells.

PBMCs were also stimulated with PMA/ionomycin to observe the IFN-*γ* production and CD107a expression in NKT-like cells. Results showed that the percentage of IFN-*γ* ^+^CD107a^+^ NKT-like cells in the NC group was higher than that in the PHI group (*P* = 0.008). The proportion of IFN-*γ* ^−^CD107a^+^ NKT-like cells in the PHI group was lower than that in the CHI or LTNP groups (*P* = 0.018 and *P* = 0.011). The percentage of IFN-*γ* ^+^CD107a^−^ NKT-like cells in NC group was much higher than HIV-infected groups (PHIs, CHIs, and LTNPs) (*P* = 0.014, *P* = 0.007, and *P* = 0.007, resp.) (Figure 4(b)). It indicated that NC group had strong potential abilities for IFN-*γ* production.

### 3.3. Correlation of NKT-Like Cell Function with CD4^+^ T Cell Counts and Viral Loads in HIV-Infected Individuals

**Figure 5:**
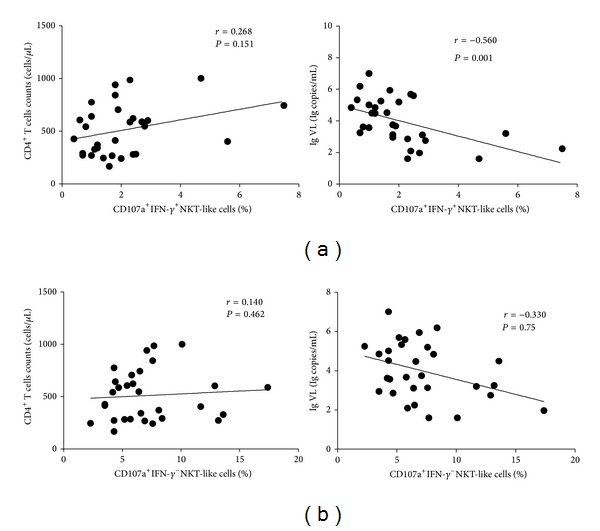
Correlation of NKT-like cell function with CD4^+^ T cell counts and viral loads in HIV-infected individuals. (a) When NKT-like cells in PBMCs were cultured together with the MHC null K562 cell line, we analyzed the correlation of the levels of IFN-*γ* ^+^CD107a^+^ NKT-like cells with CD4^+^ T cell counts and viral loads. (b) With PMA and ionomycin stimulation, we analyzed the correlation of the levels of CD107a^+^IFN-*γ* ^−^ NKT-like cells with CD4^+^ T cell counts and viral loads. Correlations between variables were evaluated using the Spearman's rank correlation test and *P* values <0.05 were considered significant.

We analyzed the relationships between NKT-like cell functions and CD4^+^ T cell counts or viral loads. When NKT-like cells in PBMCs were cultured together with the MHC null K562 cell line, it showed that the percentage of IFN-*γ* ^+^CD107a^+^ NKT-like cells did not relate to the CD4^+^ T cell counts (*r* = 0.268, *P* = 0.151). However, the percentages of IFN-*γ* ^+^CD107a^+^ NKT-like cells negatively correlated with viral loads (*r* = −0.560, *P* = 0.001) (Figure 5(a)). Percentages of CD107a^+^ NKT-like cells negatively correlated with viral loads (*r* = −0.669, *P* = 0.000) (data not shown).

When NKT-like cells in PBMCs were coincubated with the strong stimulation of PMA/ionomycin, no correlations were established between percentage of IFN-*γ* ^−^CD107a^+^ NKT-like cells and CD4^+^ T cell counts (*r* = 0.140, *P* = 0.462) or viral loads (*r* = −0.330, *P* = 0.75) (Figure 5(b)). The percentage of CD107a^+^ NKT-like cells exhibited a negative correlation with viral loads (*r* = −0.363, *P* = 0.049) (data not shown).

## 4. Discussion

Components of the innate immune system known as NK cells, iNKT cells, CD3^+^CD56^+^ NKT-like cells, and other cells make up part of the first line of defense in the immune system. These cells play an important role in inhibiting viral infection and controlling tumors through secretion of interleukins (ILs), Interferons (IFNs), and chemokines [16, 17]. NKT like cells are different from classical invariant NKT (iNKT) cells. iNKT cells are CD1d-restricted V*α*24/V*β*11^+^ *α*-galactosylceramide (*α*-GalCer)-reactive T cells, while NKT-like cells are CD1d-independent and express diverse T-cell receptors (TCRs) [18, 19]. CD3^+^CD56^+^ NKT-like cells may serve as an early source of regulatory cytokines, such as IFN-*γ* and IL-2 [20, 21]. In addition, Bamias et al. found that CD3^+^CD56^+^ NKT-like cells were significantly higher in tumor ascites compared to blood and control ascites, that higher tumor grades were associated with reduced levels of CD3^+^CD56^+^ NKT-like cells, and that the selective accumulation of CD3^+^CD56^+^ NKT-like cells in ascites may be a predictive factor for platinum resistance [22]. Fu et al. have reported that the percentage of CD3^+^CD56^+^ NKT-like cells increased in individuals after HIV-1 infection. They also found alterations of the NK cell receptors on these cells in HIV-1-positive children infected via mother-to-child transmission [23]. However, little is known about the functional activities of NKT-like cells during HIV infection.

In our study, we examined the functions of CD3^+^CD56^+^ NKT-like cells. When PBMCs were cultured with the MHC null K562 cell line, the percentage of IFN-*γ* ^+^CD3^+^CD56^+^ NKT-like cells was higher in LTNPs than that in CHIs, PHIs, or NCs. IFN-*γ* is believed to display direct anti-viral activity, promote Th1 polarization, and facilitate specific cytotoxicity by overexpression of MHC class-I and class-II molecules, antigen processing, and immunoglobulin switching [24, 25]. During HIV infection, IFN-*γ* directly counters HIV infection by inhibiting HIV tat-protein-mediated long terminal repeat (LTR) transformation [26]. Moreover, IFN-*γ* is the key factor in the activation of acquired immune responses and may be an important mechanism of resistance to HIV infection [27]. Montoya found that production of IFN-*γ* by PMA/ionomycin-activated CD3^+^CD56^+^ cells was significantly higher in ESN than in controls, which indirectly supports our findings [15]. Therefore, LTNPs displaying uniquely high levels of IFN-*γ* in NKT-like cells could offer new insights into how the immune system controls the spread of HIV.

Previous studies only focused on a single functional characteristic of CD3^+^CD56^+^ NKT-like cells. It is essential to perform a combined analysis of CD3^+^CD56^+^ NKT-like cell function to arrive at a more comprehensive description of the status of CD3^+^CD56^+^ NKT-like cell function. It was interesting to discover that there was a higher percentage of IFN-*γ* ^+^CD107a^+^ NKT-like cells in LTNPs, which was much higher than in CHIs. Viral load and CD4^+^ T cell count detection are important indicators for monitoring disease progression in HIV-infected individuals. We found that the percentage of IFN-*γ* ^+^CD107a^+^ NKT-like cells negatively correlated with viral loads. This negative correlation suggests that CD3^+^CD56^+^ NKT-like cells may inhibit viral replication and delay disease progression through IFN-*γ* secretion and degranulation.

We observed that the cytokine production and degranulation of NKT-like cells in the PHI group decreased a lot. Stimulating with the MHC null K562 cell line or PMA/ionomycin, the percentage IFN-*γ* or CD107a in CD3^+^CD56^+^ NKT-like cells were much lower in PHIs than in NCs, CHIs, or LTNPs. We speculate that innate immunity, as the first line of defense, must exert a fierce anti-HIV activities in primary infection, and this response may lead to a temporary depletion of cellular functions. The blood of primary HIV infected individuals was reported to contain an intense, proinflammatory cytokine “storm,” which was followed by immunoregulatory cytokine production [28]. During primary HIV infection, the significant reduction in intracellular cytokine levels might be due to cytokines being released from cells into the bloodstream. Lower IFN-*γ* production and CD107a expression by CD3^+^CD56^+^ NKT-like cells could suggest that the innate immune system plays an important role in primary infection and lead to the temporary inactivation of IFN-*γ* production and CD107a expression. Innate immune function may be restored in the chronic phase of infection. Golden-Mason et al. described phenotypic and functional changes of cytotoxic CD3^+^CD56^+^ NKT-like cells that determined the outcomes of acute hepatitis C infections. They also found reduced numbers of IL-2- and IL-13-producing CD3^+^CD56^+^ NKT-like cells in acute HCV infections, indicating that the function of innate immune cells is depleted in the primary stage of HCV infection [29]. This functional depletion indirectly supports our speculation about temporary cell function depletion. Researchers suggested that the cytotoxic function of CD3^+^CD56^+^ NKT-like cells may correlate with the level of MHC class I chain-related molecules (sMICs) and with natural killer group 2D (NKG2D) expression on CD3^+^CD56^+^ NKT-like cells [8]. A comprehensive investigation of the expression of inhibitory and activating NK-associated receptors (NKRs) on CD3^+^CD56^+^ NKT-like cells would be a good topic for future research.

## 5. Conclusion

Our results showed that the percentage of IFN-*γ* ^+^CD3^+^CD56^+^ NKT-like cells was obviously higher in LTNPs than in CHIs, and the proportion of IFN-*γ* ^+^CD107a^+^ NKT-like cells in LTNPs was much higher than in CHIs. Additionally, percentages of IFN-*γ* ^+^CD107a^+^ NKT-like cells negatively correlated with viral loads. These results suggest that large amounts of IFN-*γ* production by CD3^+^CD56^+^ NKT-like cells in LTNPs may be one factor involved in controlling HIV infection. Therefore, we conclude that CD3^+^CD56^+^ NKT-like cells possess functional activity that counters the progress of HIV infection and that these changes likely occur as a protective mechanism to limit viral spread and help slow HIV progression to AIDS in HIV-infected individuals.

## Figures and Tables

**Table 1:** Characteristic of study subjects.

Patient group	Number of individuals	CD4 count (range)	Viral load count (range)
Primary HIV infection (PHI)	10	484 (166–774)	52,000 (728–10,000,000)
Chronic HIV infection (CHI)	13	328 (240–986)	30903 (40–489779)
Long-term nonprogressor (LTNP)	7	704 (547–1000)	562 (40–5623)
Normal control	14	N/A^a^	N/A^a^

N/A^a^: not available.

## List of Abbreviations

NKT-like cell: Natural killer T-like cell
HIV: Human immunodeficiency virus
HAART: Highly active antiretroviral therapy
PHI: Primary HIV-infected individual
CHI: Chronic HIV-infected individual
LTNP: Long-term nonprogressor
NC: Normal control
IFN-*γ*: Interferon-*γ*
iNKT: Invariant NKT
NK: Natural killer cell
MHC: Major histocompatibility complex
HLA: Human leukocyte antigen
HCV: Hepatitis C virus
CLL: Chronic lymphocytic leukemia
ESN: Exposed HIV-seronegative individuals
PBMC: Peripheral blood mononuclear cell
IL: Interleukin
LTR: Long terminal repeat
sMIC: MHC class I chain-related molecule
NKG2D: Natural killer group 2D
NKR: NK-associated receptor
AIDS: Acquired immune deficiency syndrome.
